# Bats Swarm Where They Hibernate: Compositional Similarity between Autumn Swarming and Winter Hibernation Assemblages at Five Underground Sites

**DOI:** 10.1371/journal.pone.0130850

**Published:** 2015-07-08

**Authors:** Jaap van Schaik, René Janssen, Thijs Bosch, Anne-Jifke Haarsma, Jasja J. A. Dekker, Bart Kranstauber

**Affiliations:** 1 Department of Behavioural Ecology and Evolutionary Genetics, Max Planck Institute for Ornithology, Eberhard-Gwinner-Strasse, 82319, Seewiesen, Germany; 2 Bionet Natuuronderzoek, Valderstraat 39, 6171EL, Stein, The Netherlands; 3 Ad Hoc Zoogdieronderzoek, Oude Velperweg 34, 6824HE, Arnhem, The Netherlands; 4 Animal Ecology & Ecophysiology group, Institute for Water and Wetland Research, Radboud University Nijmegen, P.O. Box 9010, 6500GL, Nijmegen, The Netherlands; 5 Dutch Mammal Society, P.O. Box 6531, 6503GA, Nijmegen, The Netherlands; 6 Department of Migration and Immuno-ecology, Max Planck Institute for Ornithology, Am Obstberg 1, 78315, Radolfzell, Germany; 7 University of Konstanz, Department of Biology, Konstanz, Germany; University of Western Ontario, CANADA

## Abstract

During autumn in the temperate zone of both the new and old world, bats of many species assemble at underground sites in a behaviour known as swarming. Autumn swarming behaviour is thought to primarily serve as a promiscuous mating system, but may also be related to the localization and assessment of hibernacula. Bats subsequently make use of the same underground sites during winter hibernation, however it is currently unknown if the assemblages that make use of a site are comparable across swarming and hibernation seasons. Our purpose was to characterize the bat assemblages found at five underground sites during both the swarming and the hibernation season and compare the assemblages found during the two seasons both across sites and within species. We found that the relative abundance of individual species per site, as well as the relative proportion of a species that makes use of each site, were both significantly correlated between the swarming and hibernation seasons. These results suggest that swarming may indeed play a role in the localization of suitable hibernation sites. Additionally, these findings have important conservation implications, as this correlation can be used to improve monitoring of underground sites and predict the importance of certain sites for rare and cryptic bat species.

## Introduction

Between August and October many temperate-zone bat species in both the new and old world gather at underground sites in a behaviour known as swarming [1]. The assembled bats display intense flight activity, circling in and around the entrance of the site throughout the night, but predominantly do not roost there during the day [2]. Swarming activity is largely limited to species that make use of underground sites seasonally, hibernating there in winter but roosting elsewhere in summer [3]. Many behavioural and genetic studies have shown that swarming acts as a promiscuous mating behaviour, facilitating gene flow between otherwise isolated summer colonies (eg. [4, 5, 6]). In addition, its occurrence at underground sites has often led to the suggestion that swarming also plays an important role in the assessment of the suitability of a hibernaculum [1, 7, 8] and/or the social transfer of information regarding its location [9].

Within the swarming season there is considerable variation in the timing of swarming activity among species, as well as local variation in timing based on altitude [10, 11] and latitude [12, 13]. However, the general pattern is similar for all species. The assemblage of bats is strongly male-biased, with observed sex ratios of around 4:1 [11, 12, 14], and male bats roost closer to the site than females [15]. Genetic analysis has shown that females from multiple colonies make use of a swarming site, both throughout the swarming season and on individual nights [eg. 4]. In *Myotis nattereri*, it has been shown that females from a single colony attend multiple swarming sites, with most individuals swarming at the nearest site [14]. Ringing studies suggest that individuals of both sexes display general site fidelity within and across years, being recaptured most often at the same site [16]. Similarly, individuals radio-tracked at swarming sites were never found to visit other sites [14, 17], although ringed individuals have been found at other sites [eg. 1]. Approximately one to two months after the peak swarming activity of a species, individuals return to underground sites to hibernate [3].

Whether bats have (species-specific) preferences for particular underground sites has been investigated both during swarming and hibernation seasons. Glover & Altringham [13] showed that swarming intensity was highest at underground sites with 1) extensive chamber development, 2) without hydrological activity, and 3) with a sheltered, horizontally oriented entrance. Randall and Broders [18] also found chamber length and development to be important, while also suggesting that stream length in the surrounding area positively influences swarming behaviour. Contrary to Glover and Altringham [13], Randall and Broders also found that sheltered entrances negatively influenced swarming, although all sites in their study were considered highly sheltered (21 of 25 were more than 75% sheltered). Finally, Johnson et al. [19] found that swarming bats preferred sites with a single isolated entrance, while also suggesting that a larger cave entrance size was important for several species.

During hibernation, bats have similarly been shown to have preferences for certain sites and microclimates [20, 21]. Particularly, areas with adequate and stable humidity which reduce evaporative water loss appear to be critical [22, 23]. Additionally, broad genus and species-specific preferences for temperature have been reported [24]. However, species are often found hibernating at a wide range of temperatures, and temperature preferences may also be sex and age specific [25], vary throughout the season [26–28], or vary depending on the amount of fat reserves possessed by individuals [29]. Moreover, several monitoring efforts have reported that bats frequently move within and between sites throughout the winter [21, 30], especially following extreme weather changes, suggesting they may be flexible in relocating to more optimal conditions/locations [31, 32].

Several studies have made inferences as to whether individuals observed at a site during the swarming season are also found there during hibernation (eg. whether preferences for particular swarming and hibernation sites are linked). Fenton [1] suggested that bats tended to hibernate where they had been caught during the swarming season. Likewise, Rivers *et al*. [14] suggested that at least a proportion of individuals remain at a site to hibernate and proposed that characterizing the swarming assemblage may be a suitable alternative for winter surveys, especially in the case of crevice-dwelling bats such as *M*. *nattereri*. More recently, Randall and Broders [18] explicitly made the assumption that sites used during swarming were likely to also be hibernacula. Conversely, in several studies no clear relation has been found between swarming and hibernation assemblages suggesting that bats do not exclusively hibernate where they swarm [2, 7], whereas others found generally comparable assemblages but far fewer bats during the hibernation season suggesting that most bats do not use the same site in winter [33]. Finally, several studies investigating which site characteristics are important to swarming bats have concluded that their observations are insufficient to draw clear inferences concerning the use of these sites as hibernacula [13, 19].

Here we characterize the autumn swarming activity at six known hibernacula, and subsequently compare the swarming and hibernating assemblages at five of these sites for which hibernation survey data was available. To do so, we first confirmed that the number of bats, per species, was distributed non-uniformly across the investigated sites during both seasons. We predicted that the distribution of bats among sites should be non-uniform as a result of species-specific site preferences [eg. 13, 18, 20, 27] during both swarming and hibernation seasons. Next, we assessed whether there was a statistical correlation between the observed swarming and hibernation assemblages in two different ways. Per site, we compared the composition of the swarming and hibernation assemblages by comparing the relative abundance of the different bat species between seasons. Additionally, per species, we compared the relative proportion of an individual bat species encountered at each site during the swarming and hibernation seasons. Based on previous research [1, 14, 18], we hypothesized that the preferences for particular sites during both swarming and hibernation seasons are linked, and thus we expected a compositional similarity in the swarming and hibernation assemblages, both per site and per species.

## Materials and Methods

### Study site

The study was carried out at six of the 136 disused limestone mines available to bats in the Zuid-Limburg region of the Netherlands (Table 1). These mines, together with similar mines in Belgium, are the main potentially suitable underground sites in the area, and are therefore highly important for regional bat populations. Given that the sites we surveyed are disused mines, with far fewer large cracks and crevices than most other (natural) underground sites, hibernation survey data for these mines is comparatively accurate. Nevertheless, counts likely remain an underestimate of the true number of bats hibernating at these sites [34]. The maximum pairwise distance between sites was less than 10 km (1.9–8.4 km; Table 1), well within the migratory distance of all bat species investigated [16]. Therefore we expect the selected sites to have largely overlapping catchment areas, and thus we argue that any observed differences in bat assemblage are not purely the result of differences in the local bat communities near each site. Sites were selected based on previous capture data in an attempt to get a representative sample of all species present in the area.

**Table 1:** Descriptive characteristics of the underground sites we sampled.

Location			Entrance				Inner characteristics			Species	
Site	Coordinates	Alt	N	Size (m^2^)	Orientation	Shelter	Area	Chamber	Hydr	S	H
Barakkengroeve	50.865, 5.791	99	1	4.6	Horizontal	Sheltered	33.1	Yes	Dry	10	7
Koelenbosch-groeve	50.852, 5.775	90	1	8.9	Horizontal	Sheltered	7.6	Yes	Dry	10	5
Riesenberg-Noord	50.798, 5.745	105	2	5.6	Horizontal	Sheltered	4.5	No	Dry	11	7
Groeve de Schark	50.828, 5.678	85	3	24.6	Horizontal	Exposed	3.6	Yes	Dry	10	6
Schenkgroeve	50.873, 5.766	65	2	51.6	Horizontal	Sheltered	5.9	No	Dry	8	7
Oudberggroeve	50.825, 5.665	75	1	0.09	Horizontal	Sheltered	8	No	Dry	9	-

Abbreviations: latitude and longitude in decimal degrees (Coordinates); altitude at the site entrance in meters (Alt); the number of entrances (N); the total entrance size (Size); the orientation of the entrance (Orientation); degree of shelter around the entrance (Shelter); the surface area of the mine in ha (Area); degree of chamber development (Chamber); hydrological activity in the mine (Hydr.); and the number of species observed during swarming (Swarm), and hibernation surveys (Hiber). All subjective mine characteristics (Orientation, Shelter, Chamber development, Hydrology) were characterized as described in Glover and Altringham [13]. Remaining measurements were obtained from the Studiegroep Onderaardse Kalksteengroeven [36], and updated based on the dataset of AJH.

### Swarming captures

Bats were caught using mist-nets at the entrance to each mine using a standardized net configuration for each mine. Each site was sampled eleven times, once every seven days, throughout the swarming season (from August 2, 2008 to October 12, 2008). In the event of heavy rain, sampling was carried out on the following day. For each individual bat, forearm length, body mass, sex, reproductive status, and age (adult/juvenile; based on ossification of the epiphyseal joints [35]) were recorded. Bats were temporarily marked on the toenails using non-toxic nail polish to exclude recaptured individuals. Bat captures were carried out under license from the Dutch ministry of Economic affairs, (permit FF/75A/2003/150), and with permission of all site owners (Staatsbosbeheer; Limburgs Landschap). All bats were released within one hour, at the point of capture.

### Winter hibernation surveys

Winter hibernation counts were carried out by volunteers at five of the six sites (Oudberg is inaccessible) and survey data were obtained from the Dutch Mammal Society database. Each hibernaculum was surveyed on a single day by teams with more than 10 years of surveying experience. Species were identified in situ, and bats were not handled. Ceilings of mines are low enough for reliable visual inspection, in some cases aided by binoculars. The species *M*. *mystacinus* and *M*. *brandtii* were combined, as they cannot be reliably distinguished in winter without disturbance. In cases where individuals could not be identified they were classified as “indet.” and not included in this analysis (2–12 bats per site). Survey data were available between 1994–2009; since counts for each site were comparable between years, we performed the analysis using the count data from the most recent winter season available per site (2008 for Riesenberg-Noord, 2009 for all other sites).

### Statistical analysis

Temporal patterns within the swarming season were explored visually by plotting the abundance of each bat species per week relative to the total number of bats caught in that week using R 3.1.2 (38).

To test for a disproportionate distribution of the number of bats per species across the sampled sites, we performed a Pearson's Chi-squared test in R 3.1.2 [37] within each season (cumulative swarming season and winter hibernation survey).

For the comparisons between swarming and winter assemblages, all species for which there were less than 10 winter records were omitted (*Myotis bechsteinii*, *Plecotus auritus*, *Eptesicus serotinus*). To compare the swarming and hibernation datasets, we compared two types of relative abundances rather than actual count data. This allowed us to compensate for variation in species phenology during the swarming season and to compare datasets of swarming and winter counts, which cannot be compared in terms of absolute quantities. First, per site, we calculated the relative abundance of each bat species for both the cumulative swarming season and the winter hibernation survey. Second, per species, we calculated the relative proportion of a species that was encountered at each site, again for both the cumulative swarming season and the winter hibernation survey. In both cases, the compositional similarity between the swarming and hibernation seasons was measured using a permutational analysis of variance using Bray-Curtis distance matrices (calculated using the adonis function of R package ‘vegan’ version 2.2–1 [38]). For both tests, 100 permutations were used due to the limited number of possible permutations given the size of the dataset.

## Results

### Swarming captures

**Fig 1:**
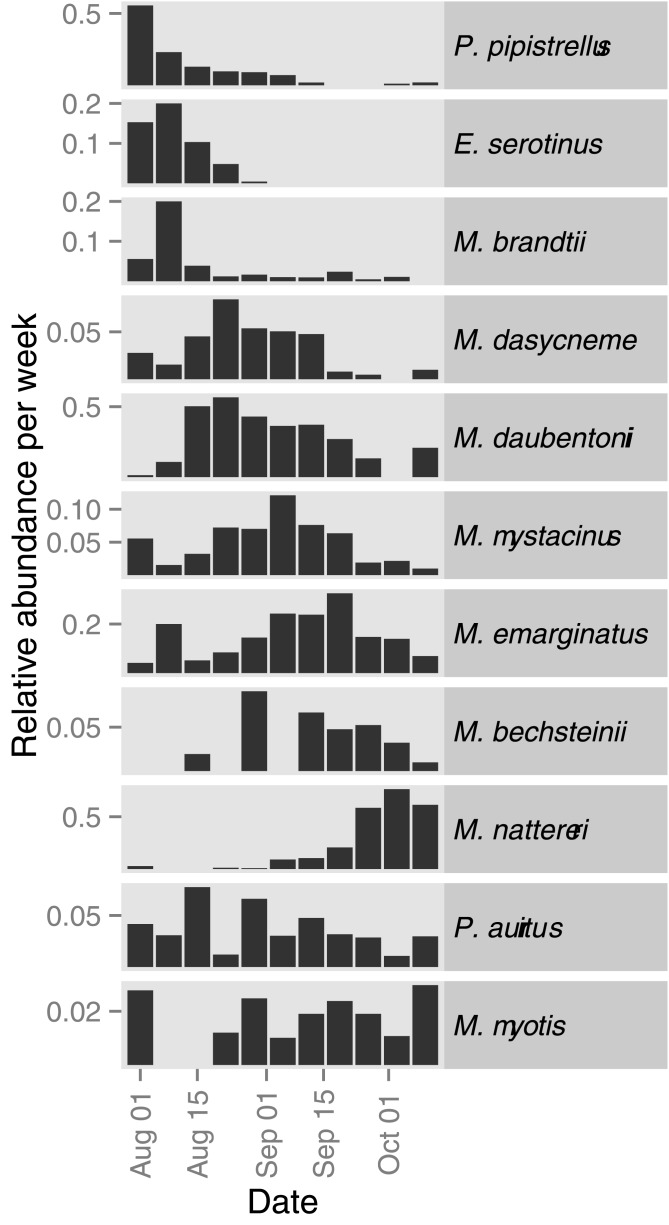
Weekly relative abundance of each species throughout the swarming season. Samples were pooled across all sampled swarming sites per week to compensate for weather effects. Species are sorted based on the timing of peak swarming activity (from earliest: *Pipistrellus pipistrellus* to latest: *Myotis nattereri*). No clear peak in swarming activity was observed for *Myotis myotis* and *Plecotus auritus*.

A total of 1351 unique individuals of 13 species were caught during this study. For two species (*Nyctalus noctula*, *Plecotus austriacus*) only one individual was caught, and therefore these species were not included in further analysis. In nine of the remaining eleven species the sex ratio was male biased (67 to 87%; Table 2), whereas two species (*Myotis myotis*, *Pipistrellus pipistrellus*) showed a slight female bias (43 and 45% males respectively). The timing of peak swarming activity varied between species (Fig 1) with *Pipistrellus pipistrellus*, *Eptesicus serotinus*, and *Myotis brandtii* peaking earliest (early August), *Myotis dasycneme*, *Myotis daubentonii*, *Myotis mystacinus* and *Myotis bechsteinii* peaking between late August and early September, and *Myotis emarginatus* and *Myotis nattereri* showing the latest peak (end of September). Two species, *Plecotus auritus* and *Myotis myotis*, did not show a peak and were found at low levels throughout the sampling period.

**Table 2:** Number of individuals captured per species and site during the autumn swarming survey.

Species	% Male	Barakken-groeve	Koelenbosch groeve	Riesenberg-Noord	Groeve de Schark	Schenk-groeve	Oudberg-groeve	Total
*Eptesicus serotinus*	87	12	12	3	13	5	0	45
*Myotis bechsteinii*	83	0	1	1	0	0	51	53
*Myotis brandtii*	86	3	24	1	0	3	4	35
*Myotis dasycneme*	71	6	12	14	0	9	1	42
*Myotis daubentonii*	76	102	80	56	25	59	68	390
*Myotis emarginatus*	82	27	64	30	3	57	25	206
*Myotis myotis*	45	10	0	5	0	8	0	23
*Myotis mystacinus*	73	32	27	0	4	1	3	67
*Myotis nattereri*	67	88	30	22	27	120	18	305
*Pipistrellus pipistrellus*	43	26	4	3	77	4	3	117
*Plecotus auritus*	81	13	4	8	13	16	2	56

Table includes the observed sex ratio for each species (% male). A single individual of *Nyctalus noctula* (Riesenberg-Noord), and *Plecotus austriacus* (Groeve de Schark) were also captured (not shown).

### Hibernation counts

During hibernation counts (January 2009; 2008 for Riesenberg-Noord), 1797 bats of eight species and one species complex were recorded. *Myotis bechsteinii* was not found at any of the sites, and both *Eptesicus serotinus* and *Plecotus auritus* were found in low numbers (1 and 9 respectively). For all sites, the number of species found during the swarming season was higher than observed during hibernation counts (Table 1).

### Distribution across sites

The distribution of bats, per species, across the investigated sites was significantly different from a uniform distribution during both the swarming (χ^2^ test, p < 0.001) and hibernation season (χ^2^ test, p < 0.001).

**Fig 2:**
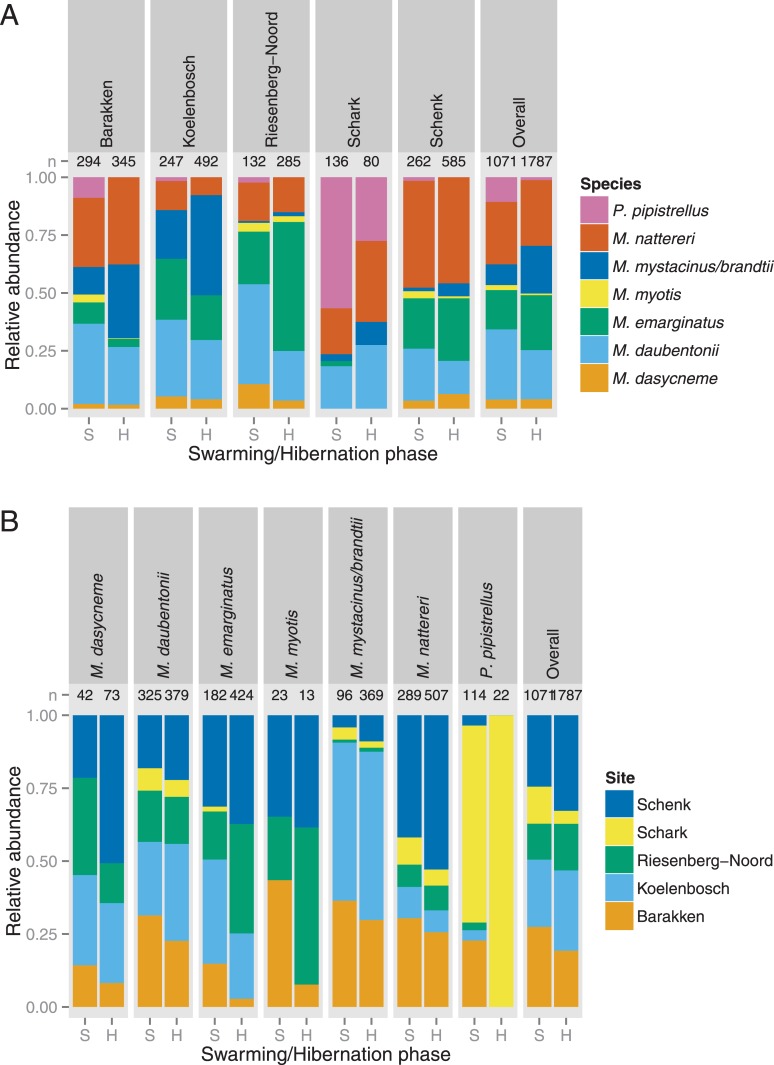
Comparison of the relative abundance of bat species during swarming and hibernation seasons measured (A) per site, and (B) per species. For both comparisons the cumulative swarming season (S) is compared to the hibernation survey (H).

For the six species and the *Myotis mystacinus/brandtii* complex, for which data in both seasons were sufficient, the per site relative abundance of a species during the swarming season was significantly correlated with the relative abundance of that species at that site during hibernation (F_4,5_ = 11.67; p = 0.02; Fig 2a). Similarly, per species, there was a clear correlation between the relative proportion of a species found at each site during the swarming season and the relative proportion of that species found at the same site during hibernation (F_6,7_ = 7.78; p = 0.01; Fig 2b).

## Discussion

The overall distribution of bats across the underground sites investigated in this study was not uniform in both seasons. As the typical migration distance of the observed species far exceeds the spatial proximity of the sites, and a large number of bats visit these sites (and the others around them), this large deviation from a uniform distribution across sites cannot only be explained by confounding factors such as landscape characteristics, or the distribution of maternity colonies in the area. Instead, in accordance with previous studies, we argue that species preferentially visit specific sites during both seasons. Indeed, we find that these site preferences appear to be linked as the relative abundance of bat species at individual sites was strongly correlated between swarming and hibernation seasons. Similarly, the relative proportion of individual bat species found at each of the five investigated sites was correlated between the swarming and hibernation seasons. The concordance between these two analyses suggests that there are no significant site- or species-specific differences in either sampling accuracy or behaviour. Taken together, these results indicate that underground sites may be actively selected by bat species, either on the basis of their suitability as swarming sites (eg. arena characteristics) or hibernation sites (eg. microclimate), or a combination of both.

Given that we do not have individualized data, we cannot determine if these correlations are the result of individual bats making use of the site during both seasons, or whether the absolute number of bats using a site in each season is comparable. In fact, several ringing and monitoring studies suggest this is not always the case [14, 16]. Nevertheless, several important conclusions can be drawn based on our observations.

### Species-specific observations

We found a male bias (> 66%) in all but two species (*Pipistrellus pipistrellus*, *Myotis myotis*), and additionally did not find a peak in swarming activity for *M*. *myotis*. Intriguingly, these two species are among those known to have other mating strategies (*P*. *pipistrellus*: songflight and male territories [39]; *M*. *myotis*: temporary harems [40]), suggesting that their behaviour at swarming sites may not be entirely analogous to that of the remaining species.

Several species were omitted from the analyses due to insufficient (<10) winter records (*Eptesicus serotinus*, *Plecotus auritus* and *Myotis bechsteinii*), or included but only found at a single site in winter (*Pipistrellus pipistrellus*). Nevertheless, these species were present in larger numbers during the swarming season. Both *E*. *serotinus* and *P*. *pipistrellus* are considered to hibernate mostly in cold crevices [41], and may have therefore been underestimated in winter due to their roosting ecology. Regarding *P*. *auritus*, we did not capture more than 16 individuals at any given site and did not find a clear activity peak during the swarming season as found in other studies [15], suggesting that the sites selected in this study may simply not be very important for this species. Lastly, *M*. *bechsteinii* only occurred in large numbers at a single site during the swarming season (Oudberg). However, as this site is inaccessible in winter, it was omitted from the comparison between swarming and hibernation assemblages. Thus, although these four species were omitted from the comparison, we believe that the observed correlations may indeed hold for these species as well.

### Function of swarming

The observed correlation between swarming and hibernation assemblages lends credibility to the theory that swarming behaviour may play a role in the inspection of, or social information transfer regarding, the suitability of an underground site as a hibernaculum in addition to its role in the mating system of these bat species.

By comparing swarming behaviour in bats to the large range of mating systems seen in another mammalian clade, ungulates, a general scenario that combines both functions can be generated. As in several ungulate systems [42], male bats of the species investigated in this study are generally unable to directly defend females or female ranges, and therefore must resort to assembling at locations common to many female groups (in bats: hibernacula). In the event that female bats come to the underground sites to (re-)inspect their suitability as a hibernaculum, the assembled males could form very small territories in these areas similar to the clustered mating territories seen in puku (*Kobus vardoni*; [42]). If, however, the females come to the underground sites solely for the purpose of mating, the assembly would resemble a classic lek (in which the females receive no benefit from visiting the site other than mating; see [43]), as seen in ungulates such as fallow deer (*Dama dama dama* [44]) and Uganda kob (*Kobus kob thomasi* [45]). In the case of a classic lek, the correlation between swarming and hibernation assemblages can still be explained, as in this scenario males must select a display habitat known to, and regularly frequented by, large numbers of females. Since the males of most swarming species are solitary or live in small male groups throughout the summer [46] and in some cases even geographically isolated from female home ranges (*M*. *daubentonii*: [47, 48]; *M*. *dasycneme*: [49]), the underground sites used communally by both sexes during hibernation would be the only such location.

Given that large numbers of juvenile bats are also caught at swarming sites, the use of swarming behaviour as a classic lek appears unlikely, as these individuals are generally not yet sexually mature. In combination with the observed compositional similarity between swarming and hibernation assemblages, we therefore suggest that male bats of these species are forming clustered mating territories at hibernacula during the autumn swarming season. However, further studies investigating the behaviour of juvenile individuals as well as individualized studies on the visitation patterns of adult individuals will be required to further elucidate the function of swarming behaviour in bats.

### Conservation applications and implications

Our findings support previous suggestions that the swarming assemblage may be an informative proxy for relative population size and importance of an underground site during hibernation [14]. However, it is important to take into account that sampling must take place throughout the entire swarming season to sample all species, and that inferences regarding the relative importance of a site as compared to others in the area will require similarly detailed information for surrounding sites. Nevertheless, when designed correctly, such approaches can aid greatly in the identification and protection of important underground sites, especially when applied to species-specific research objectives. For example, several species such as *M*. *nattereri* and *M*. *bechsteinii* often hibernate in deep crevices and karst formations [50]. By surveying potential hibernacula during the swarming season it may be possible to identify the most important underground sites for such elusive species. Notably, this correlation can also be used to predict the importance of underground sites that are too dangerous or too difficult to access in winter (eg. the Oudberg in this study). Similarly, for species that are indistinguishable in winter (eg. *M*. *mystacinus* / *M*. *brandtii* / *M*. *alcathoe*), swarming surveys can provide an indication as to the relative contributions of each of the cryptic species. Finally, recent advances in remote monitoring technologies (eg. camera-trap logging [50]), may further simplify and enhance the conservation applications of these findings, as they will allow such studies to be carried out without invasive mist-netting and/or hibernation surveys.

These findings also have several notable implications for conservation. The non-uniform distribution of species across sites emphasizes that individual underground sites may differ greatly in terms of their importance to the local bat community. Therefore, the disturbance/loss of a single site within an area may affect the local species differently, and even comparatively small swarming sites may be critical in terms of their conservation value for specific species. In addition, if the observed non-uniformity across sites is a consequence of preferences for particular site/entrance/microclimatic characteristics, any alteration of these sites may result in significant population declines or changes in the observed species assemblage. Similarly, the compositional similarity between swarming and hibernation assemblages highlight the importance of protecting important hibernacula throughout the year, as any disturbance in either season will likely also affect the use of the site in the other season.
